# Gut Microbiome Changes in Anorexia Nervosa: A Comprehensive Review

**DOI:** 10.3390/pathophysiology31010006

**Published:** 2024-02-02

**Authors:** Wendi Zhao, Prabhath Kodancha, Soumitra Das

**Affiliations:** 1 Department of Psychiatry, University of Melbourne, Parkville, Melbourne 3052, Australia; 2 Unit of Psychiatry, Western Health, Melbourne 3021, Australia; prabhath.kodancha@wh.org.au (P.K.); soumitra.das@wh.org.au (S.D.)

**Keywords:** anorexia nervosa, gut microbiome, microbiota, *Faecalibacterium prausnitzii*, *Roseburia inulinivorans*, *Methanobrevibacter smithii*, short-chain fatty acids

## Abstract

Anorexia nervosa (AN) remains a challenging condition in psychiatric management and its pathogenesis is not yet fully understood. An imbalance in the gut microbiota composition may contribute to its pathophysiology. This review aims to explore the link between the human gut microbiota and AN (objective 1) or refeeding syndrome in AN (objective 2). The online databases MEDLINE and PsycINFO were searched for relevant studies. A total of 14 studies met the inclusion and exclusion criteria and only answered objective 1. A total of 476 AN patients, 554 healthy-weight (HC) controls, and 0 patients with other psychiatric disorders were included. Compared to HC, there were consistently reduced abundances of *Faecalibacterium prausnitzii* and *Roseburia inulinivorans*, and increased *Methanobrevibacter smithii*, in AN patients. Changes in alpha diversity were inconsistent, while beta diversity increased in four of six studies. Our model suggests that an imbalance in gut microbiota composition leads to reduced short-chain fatty acids, contributing to a proinflammatory state in AN, which is also common in other psychiatric comorbidities. Microbial changes may also contribute to the semistarvation state through endocrine changes and altered energy utilization.

## 1. Introduction

Approximately 100 trillion micro-organisms reside in the human gut [1] and are collectively termed ‘gut microbiota’; most of them are bacteria, commonly Bacteroidetes and Firmicutes, and Actinobacteria [2]. Recent studies have represented the human gut microbiome through the analysis of stool samples, which indicate significant taxonomic variability between individuals, while core metabolic pathways remain consistent [3]. There are increasing numbers of animal models [4,5] and human studies [6,7] that support the role of the gut microbiota in human health; in particular, disease states typically observe an imbalance in the composition and function of the microbiota—termed ‘dysbiosis’. However, it is unclear whether dysbiosis is a contributing factor or a consequence of disease. As the study of the gut microbiota has been an emerging field for the last decade, there are many challenges in biological interpretations: a lack of consensus on the performance of statistical methods for microbiome data analysis, heterogeneous analysis techniques, and the influence of genetics, ethnicity, and medications on microbiota composition [8].

Anorexia nervosa (AN) remains a challenging condition in psychiatric management, as the mortality rate is the highest among psychiatric disorders and can be relapsing or chronic in its course [9,10]. Less than half of the patients (46%) fully recover from AN, and it is commonly complicated by other psychiatric comorbidities such as affective disorders (1/4 AN patients) and anxiety disorders (1/4 AN patients) [10,11]. The highly comorbid nature of AN makes it difficult to discern if any gut microbiome changes are unique to AN alone or common in other psychiatric conditions. Hence, this study is limited in that it compares AN patients with patients with other psychiatric disorders, as well as healthy populations.

The pathogenesis is multifactorial and still not fully understood since it manifests in multiple non-CNS organ systems, such as immunological and endocrine dysfunction [12]. AN has a heritability component ranging from 28 to 74% and eight significant loci implicated in AN development have been identified [12,13]. Individual traits such as anxiety, perfectionism, and obsessive-compulsivity are both risk and prognostic factors [14].

AN patients typically have chronic caloric restriction, macronutrient and micronutrient deficiencies, changing food availability, and high fibre intake [15]. Genetics, infection, and inflammation can all directly affect dysbiosis, though profound changes in gut microbiota may be a result of changes in macronutrients [16]. The interlinking of multiple variables that affect dysbiosis highlights a limitation in investigating the role of dysbiosis in AN pathogenesis, whether it contributes to the maintenance or precedes the onset of AN.

AN patients are significantly underweight and require nutritional rehabilitation that comes with the potentially fatal risk of refeeding syndrome [12]. Current identification of high-risk refeeding syndrome patients follows guidelines developed by the National Institute for Health and Clinical Excellence (NICE) [17], and another screening test is the Short Nutritional Assessment Questionnaire [18]. However, these existing strategies to identify at-risk patients remain poorly validated, especially for predicting severe hypophosphatemia, a key characteristic of refeeding syndrome [19]. There is currently no systematic review in the search for ((refeeding syndrome) AND (microbiome)) on MEDLINE and PsycINFO. The field of refeeding syndrome research is currently limited by a lack of a universally recognized definition and difficulty in diagnosis owning to its non-specific symptoms [20,21].

This review seeks to investigate the link between the human gut microbiome and AN or refeeding syndrome in AN, and whether there is scope for novel interventions to improve AN outcomes and predict refeeding syndrome development. Such interventions require substantial and validated knowledge of disease pathogenesis and the involvement of gut microbiota changes if any are proven evident. Recent systematic reviews have identified some patterns of gut microbiota alterations in AN patients [22,23,24,25], yet there remains a lack of consistent and substantial evidence of the link between the human gut microbiota and AN, or refeeding syndrome in AN. Compared to another recent review by Garcia et al., this study does not include preclinical studies or case reports; our search is limited to two databases, and compares AN to other psychiatric disorders, not only control groups [25]. PRISMA search results are limited in this field, as gut microbiota has only become of scientific interest in the last decade.

This review thus aims to critically evaluate the following research questions in the literature:

1. The qualitative and quantitative changes in gut microbiome composition in patients with AN, compared to healthy populations (HC) or patients with other psychiatric disorders;
2. The relationship between changes in gut microbiome composition and the risk of refeeding syndrome in AN patients.

## 2. Materials and Methods

A systematic search was conducted on 11 June 2023 for all articles relating to the objectives in the English language from 1949 to 2023 in the databases MEDLINE and PsycINFO. The search terms consisted of ((gut) OR (gastrointestinal) OR (intestinal)) AND ((microbiome) OR (microbiota) OR (microflora) OR (dysbiosis)) AND ((anorexia nervosa) OR (eating disorders) OR (refeeding syndrome)). 

The PICOS criteria for the inclusivity of studies are detailed in Table 1, where ‘Intervention’ was modified to ‘Investigation’. Exclusion criteria included any animal or in vitro studies, systematic reviews, case studies, conference abstracts, study protocols, and letters to the editor.

## 3. Results

**Figure 1:**
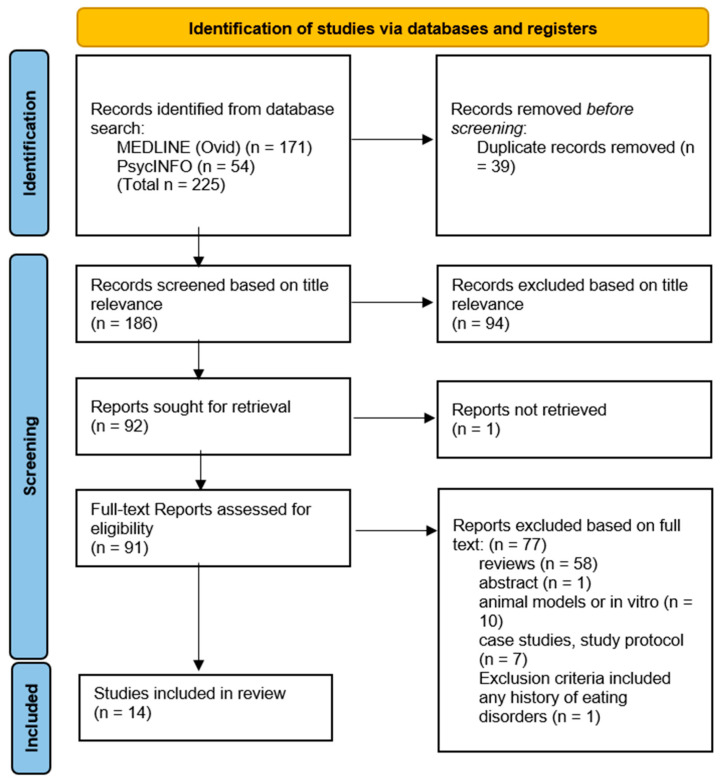
PRISMA flow diagram.

Based on the search strategies, 225 studies were initially identified (171 MEDLINE, 54 PsycINFO) and subsequently screened for inclusion in this review, as summarised in the PRISMA flow diagram (Figure 1). After 39 duplicates were removed, 186 studies were assessed on the basis of title relevance to the objectives of this review. The remaining 92 full-text studies were read and assessed based on the inclusion criteria (Table 1). The reasons for exclusion are stated in Figure 1. Ultimately, a final 14 studies remained for inclusion that answered research question 1 (n = 14), and no studies remained for research question 2 (n = 0).

Table 2 combines the data on the study design, sample size and BMI, analysis techniques, and outcomes for all studies. Key microbial changes are summarised in Table 3. Of the 14 studies, 9 were cross-sectional studies, including 1 study with a randomised control trial (RCT) component, and 5 were longitudinal studies. All studies were conducted between 2009 and 2023, and sample sizes were small–moderate (ranging from 9 to 93 AN patients): a total of 476 AN patients, 554 healthy-weight controls (HC), and 0 patients with psychiatric disorders other than AN. The criteria used to diagnose AN were a combination of DSM-4 (five studies), DSM-5 (five), EDI-2 (one), EDI-3 (one), ICD-10 (one), and unspecified criteria (Mack et al. recruited patients with a primary diagnosis of AN and admitted to an inpatient treatment program that aimed to increase body weight). Average BMI in AN patients was 14.27 kg/m^2^ and 21.51 kg/m^2^ in HC. A total of 10 studies were conducted in Europe, 2 in Asia, and 2 in America, with the vast majority of patients assessed in the setting of inpatient eating disorder units.

### 3.1. Research Question 1 Findings

#### 3.1.1. AN Patients Compared to Healthy Controls (HCs)

All included studies have demonstrated changes in gut microbiome composition in AN patients, in comparison to healthy-weight controls (HCs) (Table 2). In all four studies that detected it, there was a consistent depletion of *Faecalibacterium* abundance in AN patients’ gut microbiota compared to HCs [29,31,37,39]. Similarly, in all four studies that detected it, there was a consistent depletion of *Roseburia* abundance in AN patients’ gut microbiota compared to HCs [27,28,30,32]. There was an increased abundance of *Methanobrevibacter smithii* among AN patients compared to HCs in all four studies that detected it [26,27,32,33]. Changes in alpha diversity were inconsistent, as five studies found a significant reduction in AN compared to HCs [29,30,31,34,36], whereas five studies found no significant changes [27,32,37,38,39]. Beta diversity within the gut microbiota of AN patients was found to increase in four of the six studies that detected it when compared to HCs [28,32,37,39], while two studies found no significant changes [27,34].

#### 3.1.2. AN Patients Compared to Patients with Other Psychiatric Disorders

None of the studies used a comparator sample of patients with depression or anxiety alone, so this review was unable to directly compare AN patients to those with other psychiatric conditions. Six studies did indeed test psychological parameters (Table 4), and all four studies that tested the Beck depression inventory showed that AN patients had at least mild levels of depression (based on mean score). The following are the findings for the sample of AN patients with depression or anxiety: a negative correlation between faecal butyrate concentration and depression and anxiety scores [27]; a negative correlation between alpha diversity and levels of depression [31,36]. Both findings are the same in AN patients compared to HCs.

## 4. Discussion

### 4.1. Changes in Gut Microbiome Composition

#### 4.1.1. Alpha Diversity

In microbiology, alpha diversity estimates the diversity within a single community, comprising the number of species present (richness) and the distribution of the number of organisms per species (evenness), i.e., their relative abundance and taxonomic distribution [40]. The diversity of a community is highly related to its environment and decreases in the setting of environmental changes (e.g., from a healthy to a diseased state). For example, alpha diversity is often decreased in irritable bowel syndrome [41]. In addition to observing decreased alpha diversity in AN (Table 2), this review highlighted two studies that observed an increase in alpha diversity in post-weight-restoration patients compared to before intervention [29,34], suggesting that low microbial diversity is implicated in lower BMI and greater starvation severity in AN. However, reduced alpha diversity is not a finding specific to AN, as it is also observed in AN patients with depression or anxiety (Table 4). Depression is a prominent psychiatric feature secondary to the sequelae of semistarvation in AN [9], so it is possible that reduced alpha diversity may represent a more severe disease pathology [31]. However, there were inconsistencies in the alpha diversity changes in AN patients compared to HCs [27,32,37,38,39], and post- compared to pre-weight-restoration [31]. Differences in the size of patients’ stool samples and sample analysis techniques may explain these discrepancies. Furthermore, the discrepancy in alpha diversity data may be explained by the differences in the measurement indices (Shannon, Chao, Fischer, etc.)—there does not exist an absolute measure of diversity and each method has its own biases and advantages. Hence, the lack of specificity in choosing appropriate methods can lead to the oversimplification of diversity outcomes [42]. For example, the role of alpha diversity in general disease pathogenesis remains inconsistent, as studies in other disease states report an increase in alpha diversity [43,44].

Hence, it may be more useful to postulate the role of gut microbiota changes in AN pathogenesis by scrutinising individual taxa; however, this comes with another set of limitations. There is an inherent limitation in the interpretation of bacterial relative abundance for clinical analysis, as compositional data are not independent of each other and different biological scenarios can yield the same proportions of taxa over changes in time [45]. Reference frames have been identified as a way to alleviate false positives [45]. The reliability of compositional analysis is limited to the resolution of the sequencing method—16S rRNA sequencing or shotgun metagenomics and metagenomics relies on the known genomes of gut microorganisms [46].

#### 4.1.2. Decreased *Faecalibacterium prausnitzii*

**Figure 2:**
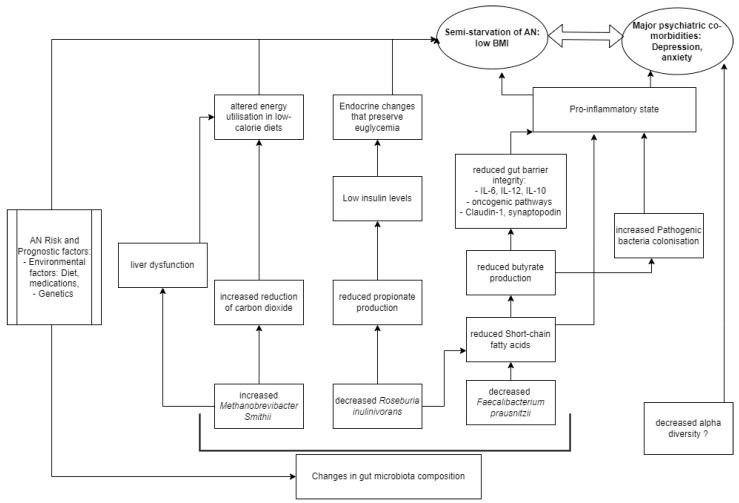
Model of AN pathogenesis related to gut microbiota.

Table 3 shows that, most significantly, AN microbial communities were consistently depleted in *Faecalibacterium* [29,31,37,39] compared to HCs, and one study found a negative correlation between eating disorder scores and *Faecalibacterium prausnitzii* [28]. Faecalibacterium metabolise dietary fibres and other complex carbohydrates to produce short-chain fatty acids (SCFAs), such as butyrate [47]. SCFAs maintain the integrity of the intestinal barrier [48], promote immune cell recruitment to the gut, and increase the production of inflammatory mediators [49]. One type of SCFA is butyrate, and *Faecalibacterium prausnitzii* is the most common butyrate-producing species in faecal samples (5% abundance in healthy stool) [47]. Butyrate maintains the immunological aspect of gut barrier integrity by regulating Claudin-1 and synaptopodin expression, limiting pro-inflammatory cytokines (IL-6, IL-12), and inhibiting oncogenic pathways [47]. The synthesis of proinflammatory IL-6 and IL-12 is inhibited through the action of *Faecalibacterium prausnitzii* components inducing the IL-10 production in immune cells [50]. In addition to maintaining healthy intestinal barrier integrity, butyrate produced by *Faecalibacterium prausnitzii* also plays an important role in restricting the entry and establishment of pathogenic microbes [51]. Butyrate activates PPAR-ɣ signalling, which drives the high-oxygen-consuming metabolism within colonocytes, maintaining a state of epithelial hypoxia [51]. The anaerobic environment in the gut lumen, maintained by butyrate-producing bacteria, prevents colonisation by pathogenic *Salmonella* and *E. coli* [52]. Hence, in all these ways butyrate normally modulates the inflammatory responses within the gut. However, where these butyrate-producing species are decreased, we hypothesize that there exists a pro-inflammatory state in the gut that contributes to AN (Figure 2).

However, we acknowledge that reduced butyrate is not a finding specific to AN alone, as it is also observed in AN patients with depression or anxiety. There is a growing body of evidence to show that depression is associated with a chronic, low-grade inflammatory response and activation of cell-mediated immunity [53], similar to our speculation on the proinflammatory state in AN pathogenesis. The disrupted host inflammatory responses may be a result of butyrate or other SCFAs, as there is evidence to support that several SCFA-producing species (*Bifidobacterium*, *Faecalibacterium*, *Lactobacillus*) are reduced in depression and anxiety [53,54]. A negative correlation was observed between *Faecalibacterium* and the severity of depressive symptoms in a cross-sectional study [53]. Hence, a reduction in *Faecalibacterium* abundance is a finding non-specific to AN compared to other psychiatric disorders, but its abundance may negatively correlate to severe psychiatric disease [28].

#### 4.1.3. Decreased *Roseburia inulinivorans*

*Roseburia*, another butyrate-producing Firmicute of similar significance in our results, was consistently depleted [27,28,30,32], and negatively correlated with Eating Disorder Examination (17th edition) scores [34]. While decreased *Roseburia* and *Faecalibacterium* remain largely responsible for the relative decrease in butyrate [27,32,37], one study in this review also observed a significant positive correlation between Roseburia inulinivorans and insulin levels in AN patients compared to HCs [27]. Levels of insulin are known to be reduced in AN patients compared to HCs, a phenomenon that helps preserve euglycemia by reducing cellular glucose uptake and glycogenesis [55], and the possible link to *Roseburia* species may be in propionate. Propionate, which is produced by *Roseburia inulinivorans* from fucose [56], has been shown to directly stimulate insulin secretion via protein kinase C and protect beta cells from apoptotic stimuli in the long term [57]. Decreased propionate levels in AN were observed in two studies [27,35] (Table 3), suggesting that the effective impacts of altered gut bacteria are endocrinological as well as neuro-inflammatory and immunological in contributing to the semistarvation state of AN (Figure 2).

#### 4.1.4. Increased *Methanobrevibacter smithii*

Another consistent alteration in AN microbiome composition is the enrichment of *Methanobrevibacter smithii* [26,27,32,33], which is already well-documented as representing an adaptive response to prolonged caloric restriction [23]. *Methanobrevibacter smithii* uses hydrogen to reduce carbon dioxide to methane, allowing for optimal nutrient transformation in very low-calorie diets [26]. However, higher levels of the archaea have also been found in obesity, constipation, and irritable bowel syndrome [58], as well as non-alcoholic fatty liver disease and cirrhosis [27]. Borgo et al. also confirmed previously described increases in liver enzymes (ALT and AST) compared to HCs [27]. Hence, it is possible that *Methanobrevibacter smithii* contributes to altered metabolism, through a disruption of liver function commonly seen in AN patients (Figure 2). The contribution of *Methanobrevibacter smithii* to the semistarvation of AN is supported by Million et al., who observed a negative correlation between BMI and *Methanobrevibacter smithii* [33].

#### 4.1.5. Comparison to Other Existing Pathophysiological Models

Gabriel et al. observed a chronic AN cohort (average illness duration 5.5 years) with a non-inflammatory cytokine profile; similarly, Nisson et al. studied a chronic AN cohort (average illness duration 10.8 years) with non-high levels of IL-6 in AN patients compared to HCs [59,60]. A meta-analysis of shorter-AN-duration cohorts showed elevated cytokine levels compared to HCs for IL-1beta, TNF-alpha, and IL-6; it appears that immune status varies according to AN disease duration [61,62]. This raises the limitation that most of the studies did not specify the time from the onset of disease, which means that the proinflammatory model in AN (Figure 2) is limited to the assumption of acute disease. In a review of genetic risk factors for eating disorders, Himmerich et al. highlighted an additional genetic role of SCFAs, as butyrate is an HDAC inhibitor with potential effects on gene expression in human cells [63].

Another hypothesis suggests that the energy needs of the gut microbiome may regulate the aberrant eating behaviour of individuals with AN; bacteria may produce modules that regulate the production of neurohormones involved in mood and eating behaviour or act directly as neurohormone-like molecules [64]. This describes a different link between SCFAs and AN, whereby SCFAs can directly act on enteroendocrine cells of the intestinal epithelium and activate the release of hormones contributing to satiation such as peptide tyrosine tyrosine or glucagon-like peptide 1 [64]. Researchers are increasingly focusing on the *Enterobacteriaceae* ClpB protein (caseinolytic peptidase B protein homologue) that can mimic the alpha-melanocyte-stimulating hormone involved in appetite control [64,65]. Three studies have found an increased prevalence of Clp-B-producing bacteria in AN patients [27,30,33].

The validity of the proposed AN model could be confirmed by successful therapy through the restoration of SCFAs (Figure 2). Systematic reviews of the last three years present similar findings to our own, pointing to decreased butyrate-producing bacteria as potential hallmarks of the gut microbiota in AN [22,24]; however, the most recent one found only inconsistent results of SCFA faecal concentrations [25]. Garcia et al. similarly found inconsistent results in alpha diversity (three studies found lower levels, and five could not replicate these results) in AN patients compared to HCs, extensively highlighting the variables that hinder the interpretation of the microbiota contribution to AN [25]. A common limitation of this review is the validity of comparison with the normal weight group for aetiological purposes, due to the high inter-individual variability among humans [25]. Hence, future studies could be designed to compare other states of malnutrition.

### 4.2. Refeeding Syndrome

Our search did not yield any non-review studies that investigated the relationship between changes in gut microbiome composition and the risk of refeeding syndrome in AN patients. However, there have been numerous systematic reviews in recent years surrounding refeeding syndrome management in AN. Although recent insights support higher calorie refeeding since it is not associated with an increased risk of refeeding syndrome [66,67,68,69], no consensus has been reached about the effect of high-fibre diets in reducing the risk. In our review, *Faecalibacterium*, *Roseburia* and other butyrate-producing Firmicutes ferment dietary fibre and are consistently depleted in AN patients relative to healthy controls (Table 3). Hence, we support the mounting postulation that gradually introducing high-fibre foods in refeeding AN patients can promote a gut microbial composition similar to that of healthy samples [70]. Six of the included studies (Table 2) observed gut microbial composition post-weight-restoration, of which two are known to provide high-calorie diets (high in protein, fat, and predominantly carbohydrate) resulting in an increase in alpha diversity compared to before refeeding [29,34]. In contrast, only one study utilised a high-fibre, high-fat, and high-energy diet that resulted in decreased Bacteroidetes and increased Firmicutes compared to before refeeding [32]. The increase in Firmicutes is unlikely to be due to the high fibre consumption as total SCFA concentrations do not differ after refeeding; rather, it is likely attributable to the high fat or carbohydrate intake [71].

Systematic reviews involving adolescents with AN have found that a lower BMI at the time of hospital admission is a better predictor of hypophosphatemia than initial calorie intake [67,72]. Hence, we postulate that a change in the relative abundance of those gut microbiota that correlate with low BMI may be associated with a higher incidence of developing hypophosphatemia: lower abundance of *Lactobacillus*, higher *Methanobrevibacter smithii*, higher *Bacteroides uniformis*, higher Verrucomicrobiaceae and Ruminococcacea, lower Clostridales, lower Turicibacteraceae, lower Eubacteriaceae, higher *Escherichia coli*, higher *Bifidobacterium animalis*, higher *Clostridium XVIII*, higher Bacteroidota, and higher Proteobacteria [26,27,30,33,34,39]. The validity of this refeeding syndrome model could be confirmed by significantly reduced or absent disease occurrence in longitudinal studies following restoration of those mentioned microbiota changes that correlated with low BMI. However, as there are evidently many bacteria possibly implicated, care should be taken to isolate the outcome corresponding to individual bacterial changes and there is a need for a specific definition of lower BMI that may predict hypophosphatemia.

### 4.3. Future Directions

There are a number of therapeutic interventions that aim to restore a healthy gut microbiome in AN patients and may supplement nutritional rehabilitation to restore weight. All six longitudinal studies that examined microbiota post-refeeding find that a healthy gut microbiota is not fully restored, and there remain significant differences in compositional diversity (Table 2). Furthermore, two of these studies demonstrated that the microbial composition of AN patients after weight gain is more similar to the individuals’ own faecal samples at admission than it is to the microbial composition of HCs [32,37]. The systematic difference between the patient and HC groups may be explained by the ‘Anna Karenina principle’, according to which dysbiotic individuals vary more in microbial community composition than healthy individuals as a stochastic response to stressors [73]. While this principle has been applied to HIV, type 1 diabetes, and Crohn’s disease [73], it can also explain the consistently increased beta diversity in AN patients compared to HCs [28,32,37,39]. Different gut bacteria may contribute to the same pathophysiology of AN, but these communities vary more compared to HC groups than to other AN patients. It follows that faecal microbiome transplantation (FMT) can be a technique to directly reduce the distance induced by health status (measured by beta diversity) and can shift the composition towards that of a healthy donor stool, though there is currently no evidence that this shift endures over time after transplantation in an AN patient [74]. There are only two studies published to date that examine the effect of FMT in AN patients [74,75] and patient clinical improvement after FMT still needs to be proven with randomised controlled trials (RCTs) to improve specificity in this field.

### 4.4. Limitations

There are several limitations in the included studies. One is that most studies did not control for diet. The Mediterranean diet is known to be associated with an increased presence of Roseburia and a low-fat, high-complex-carbohydrate diet increases *Faecalibacterium prausnitzii* [76], which has implications for our 10 European-based studies. The gut microbiome composition is strongly influenced by both long-term and short-term dietary intake [77,78]. However, AN patients in at least 10 studies resided in an inpatient unit and consumed a standard diet for a short duration. Another limitation in the study of microbial composition is that some AN participants remained on their treatment-as-usual antidepressants [36]. The antidepressants, in particular sertraline, fluoxetine, and escitalopram, are shown to have antimicrobial activity in vitro [79,80]. Additionally, microbiome composition varies between ethnicities, possibly due to variations in host genes [81]. Our study is limited to a sample of predominantly European women, mostly extremely malnourished with BMI < 15 kg/m^2^, and cannot account for mild-to-moderate severities of AN (DSM-5). Increased representation of all genders, ethnicities, and severities of malnutrition can increase the external validity of the findings in this field.

Another major limitation is the heterogeneity of analysis techniques, which may introduce bias in interpreting microbial composition results. In studies that compare 16S rRNA sequencing and shotgun metagenomic sequencing, no consensus has been reached on the superior technique for microbiome characterisation [82,83,84]. One cross-sectional study that utilised a breadth of taxonomic composition and diversity revealed that both techniques performed similarly in many aspects of bacterial community characterisation [82]. However, in comparison studies of human-gut-derived bacteria, insights favour the conclusion that shotgun metagenomics offers a higher resolution of taxonomic analyses compared to 16S rRNA sequencing [83,84]. In comparing stool samples, shotgun sequencing identified a greater number of species per sample and displays higher sensitivity compared to 16S rRNA, yet specificity is not assessed [84]. In identifying the highly complex microbial library of stool samples from Crohn’s disease patients, the pattern of relatively abundant genera is different among the two techniques, although there was significant similarity in the 16S rRNA sequences of the two genera [83]. Similarly, the highly complex microbial composition of AN patient stool samples may warrant that only those genera with significant phylogenetic differences are detected by 16S rRNA sequencing.

The methodology in this review is limited to qualitative synthesis only. There was no analysis of pooled data and quantitative synthesis, which could remove discrepancies in the heterogeneous measures of alpha and beta diversity [27,28,29,30,31,32,34,36,37,38,39]. One existing meta-analysis attempts to remove methodological heterogeneity by using a common sequencing pipeline but is limited to a few studies that use 16S rRNA sequencing [24]. While few studies in this review have a longitudinal design, the majority of studies are cross-sectional studies that undermine conclusions about causality (Table 2). Furthermore, given that our broad search did not yield any studies on the topic of refeeding syndrome and gut microbiota, the scope of research question 2 was limited and lacked enough background research to generate productive discussion.

## 5. Conclusions

There is consistent evidence to support changes in gut microbiome composition in AN patients compared to health-weight controls, yet it remains unclear whether these changes contribute to the maintenance or precede the onset of AN. No results were obtained for refeeding syndrome. Microbial community changes in AN manifest in reduced anti-inflammatory effects at the level of the intestinal barrier, implicating a pro-inflammatory state in AN (Figure 2). However, this state is non-specific to AN compared to other psychiatric disorders. Our model is limited by difficulties in microbiota interpretation, heterogeneous analysis techniques, and the impact of diet, genetics, and extreme malnourishment. Further research on AN and its comorbidities, as well as improvements in microbiome analysis, are needed to better reconcile the role of the gut microbiome in treating this complex disorder.

## Figures and Tables

**Table 1:** PICOS study inclusion criteria.

(P)opulation	Patients diagnosed with AN according to DSM-IV, DSM-V, ICD-10, EDI-3 or other, patients of both sexes and all ethnicities, aged > 12 years old and BMI < 18.5 kg/m2.
(I)nvestigation	Assessment of gut microbiota composition.
(C)omparators	HC (healthy-weight control) group or patients with other psychiatric disorders.
(O)utcomes	Analysis of the gut microbiota derived from stool samples using shotgun metagenomic sequencing or 16S rRNA sequencing techniques and/or real-time polymerase chain reaction (rt-PCR). Measures for microbiota composition in patients with AN and HC: - Relative or absolute abundance of individual phyla, genus, or species; - Alpha diversity indices; - Beta diversity indices; - Faecal metabolite concentrations (e.g., short-chain fatty acid), or - Correlations between clinical or psychopathological parameters and the microbial composition. Measures for the incidence of refeeding syndrome and its correlation with gut microbiome composition.
(S)tudy design	Cross-sectional or longitudinal studies.

**Table 2:** Data from included studies (n = 14). CS = cross-sectional, L = longitudinal, RCT = randomised control trial, HC = healthy control, PSY = sample of patients with psychiatric disorders other than AN, FMT = faecal microbiota transplantation, richness = the number of observed species, BSI = brief symptom inventory, EDE = eating disorder examination 17th edition, BDI = Beck depression inventory, STAI = state–trait anxiety inventory, BAI = Beck anxiety inventory, HAMD = Hamilton rating scale for depression. Symbols represent increased (↑), decreased (↓), or unchanged (↔).

References	Study Design	Sample	Analysis Technique for Gut Microbiome Composition	Changes in Gut Microbiome Composition in AN Patients Compared to Healthy Controls (Statistically Significant *p* < 0.05)			Changes in Gut Microbiome Composition in AN Patients Compared to Other Psychiatric Disorders	Therapeutic Interventions	Other Parameters
				Qualitative Differences in Bacteria	Quantitative Differences in Bacteria	Metabolite Concentrations			
Armougom et al. 2009 [26]	CS	9 AN (BMI 12.73 kg/m^2^ ± 1.602) 20 HC (BMI 20.68 kg/m^2^ ± 2.014) N/A PSY	qPCR	↑ *M. smithii* ↔ Firmicutes ↔ Bacteroidetes ↔ *Lactobacillus*	N/A	N/A	N/A	N/A	Positive correlation between BMI and *Lactobacillus*. Negative correlation between BMI and *M. smithii*.
Borgo et al. 2017 [27]	CS	15 AN (BMI 13.9 ± 2.1 kg/m^2^) 15 HC (BMI 22.1 ± 2.6 kg/m^2^) N/A PSY	16S rRNA qPCR	↑ Proteobacteria ↑ Enterobacteriaceae ↑ *M. smithii* ↓ Firmicutes ↓ *Ruminococcus* ↓ *Roseburia* ↓ *Clostridium* ↓ Ruminococcaceae	↔ alpha diversity ↔ beta diversity	↓ total SCFA ↓ butyrate ↓ propionate ↔ *iso*-valerate ↔ *iso*-butyrate	All 11 patients with depression (assessed by BDI) were included in the AN patient sample. All 15 AN patients had an STAI-trait score > 40, 2 of HC. 7 AN patients had an STAI-state score > 40, 1 of HC. Negative correlation between BDI depression score and *Clostridium* spp. Negative correlation between faecal butyrate concentration and depression and anxiety scores.	N/A	Negative correlation between *Bacteroides uniformis* and BMI. Positive correlation between insulin and *Roseburia inulinivorans.*
Fan et al. 2023 [28]	CS, RCT (mice studies)	77 AN (BMI 15.6 ± 2.5 kg/m^2^) 70 HC (BMI 21.8 ± 1.9 kg/m^2^) N/A PSY	Shotgun metagenomics	↑ Christensenellales ↑ *Clostridium paraputrificum* ↑ *Lactobacillus* ↑ Ruminococcaceae-enterotype ↓ Bacteroidota ↓ Actinobacteriota ↓ *Roseburia intestinalis*, *inulinivorans*	↑ beta diversity ↔ richness	↑ indoxyl sulphate	N/A	Day 21 germ-free mice with FMT from AN showed a larger initial decrease in body weight and slower weight gain over time (compared with HC FMT mice)	Positive correlation between eating disorder scores and *Clostridium* spp. Negative correlation between eating disorder scores and *Lactococcus acidophilus* and *Faecalibacterium prausnitzii.*
Fouladi et al. 2022 [29]	L	93 AN (BMI 14.6 ± 2.12 kg/m^2^) 98 HC (BMI 21.98 ± 2.13 kg/m^2^, BMI 22.56 ± 1.60 kg/m^2^) N/A PSY	Shotgun metagenomics	↓ *Bifidobacterium adolescentis* ↓ *Faecalibacterium prausnitzii*	↓ alpha diversity	N/A	N/A	Post-weight-restoration patients (compared to before): ↑ alpha diversity	Increase in the relative abundance of fermentation pathways in AN compared to HC.
Hanachi et al. 2019 [30]	CS	33 AN (BMI 11.7 ± 1.5 kg/m^2^) 22 HC (BMI 21 ± 2 kg/m^2^) N/A PSY	16S rRNA	↑ *Turicibacter* ↑ *Anaerotruncus* ↑ *Ruminococcus* ↑ *Salmonella* ↑ *Klebsiella* ↓ *Eubacterium* ↓ *Roseburia*	↓ alpha diversity ↓ richness	N/A	N/A	N/A	Negative correlation between BMI and Verrucomicrobiaceae and Ruminococcacea. Positive correlation between BMI and Clostridiales, Turicibacteraceae, and Eubacteriaceae.
Kleiman et al. 2015 [31]	L	15 AN (BMI 16.2 ± 1.5 kg/m^2^) 14 HC (BMI 21.5 ± 1.9 kg/m^2^) N/A PSY	16S rRNA	↑ Bacilli ↑ Coriobacteriales ↓ Clostridia ↓ *Anaerostipes* ↓ *Faecalibacterium*	↓ alpha diversity ↓ richness	N/A	12 AN patients had at least mild depression (BDI), and 10 AN patients had at least mild anxiety (BAI). Greater levels of depression were negatively associated with alpha diversity.	Post-weight-restoration patients (compared to before), ↔ alpha diversity (compared to HCs): ↓ alpha diversity ↓ richness	Negative correlation between alpha diversity and levels of depression, eating disorder psychopathology.
Mack et al. 2016 [32]	L	55 AN (BMI 15.3 ± 1.4 kg/m^2^) 55 HC (BMI 21.6 ± 2.0 kg/m^2^) N/A PSY	16S rRNA	↑ Firmicutes ↑ Actinobacteria ↑ Verrucomicrobia (mucin-degraders) ↑ *Clostridium* clusters I, XI, XVIII ↑ *Methanobrevibacter* ↓ Bacteroidetes ↓ *Roseburia*	↑ beta diversity ↔ alpha diversity ↔ richness	↑ Branched-chain fatty acids (isobutyrate and isovalerate) ↓ butyrate ↔ total SCFA	N/A	Post-weight-restoration patients (compared to before): ↑ beta diversity ↑ Firmicutes ↑ richness ↓ Verrucomicrobia ↓ Bacteroidetes	N/A
Million et al. 2013 [33]	CS	15 AN (BMI 13.5 kg/m^2^) 76 HC (BMI 22.4 kg/m^2^) N/A PSY	qPCR	↑ *Escherichia coli* ↑ M. smithii ↓ *Lactobacillus reuteri*, ↓ *Lactobacillus plantarum*	N/A	N/A	N/A	N/A	Negative correlation between BMI and *Methanobrevibacter smithii*, *Escherichia coli*, *Bifidobacterium animalis.* Positive correlation between BMI and *Lactobacillus reuteri.*
Monteleone et al. 2021 [34]	L	21 AN (BMI 14.6 ± 1.3 kg/m^2^) 20 AN (BMI 20.3 ± 1.4 kg/m^2^) N/A PSY	16S rRNA	↑ Bacteroidetes ↑ Actinobacteria ↑ *Weissella* ↑ *Coprococcus* ↑ Bacteroidetes-to-Firmicutes abundance ratio ↓ Firmicutes ↓ Coriobacteriales ↓ Oxalobacteraceae ↓ *Parabacterioides*	↓ alpha diversity ↔ beta diversity	↓ sugars-derived metabolites	AN patients had significantly higher BSI scores at study entry compared to at discharge. AN at study entry mean a BSI global severity index of 22.1, compared to 12 at discharge. Positive correlation with BSI scores and negative with EDE scores: *Coprococcus*, *Clostridium IV*, *Roseburia*, *Termsporobacter.*	Post-weight-restoration patients (compared to HCs): ↓ alpha diversity (↑ compared to patients before weight restoration) ↑ Leuconostocaceae ↓ Actinobacteria ↓ Coriobacteriales ↓ Catabacteriaceae ↓ *Collinsella* ↓ *Parabacteirodes* ↓ *Catabacter*	Positive correlation between EDE scores and *Bifidobacterium* and *Enterococcus*. Negative correlation between BMI and *Clostridium XVIII*.
Morita et al. 2015 [35]	CS	25 AN (BMI 12.8 ± 1.3 kg/m^2^) 21 HC (BMI 20.5 ± 2.1 kg/m^2^) N/A PSY	16S or 23S rRNA -qPCR	↓ *Clostridium coccoides* ↓ *Clostridium leptum* ↓ *Bacteroides fragilis* ↓ *Streptococcus* ↓ *Lactobacillus plantarum*	↓ total abundance	↓ acetate ↓ propionate	N/A	N/A	N/A
Morkl et al. 2017 [36]	CS	18 AN (BMI 15.29 ± 1.28 kg/m^2^) 26 HC (BMI 21.89 ± 1.73 kg/m^2^) N/A PSY	16S rRNA	↑ Coriobacteriaceae	↓ alpha diversity ↓ richness	N/A	AN patients had a mean BDI score of 21.72 and a HAMD score of 18.22. HCs had a mean BDI of 3.19, and HAMD of 2.65. Negative correlation between alpha diversity and levels of depression.	N/A	N/A
Prochazkova et al. 2021 [37]	CS	51 AN (BMI 14.4 kg/m^2^) 67 HC (BMI 21.9 kg/m^2^) N/A PSY	16S rRNA	↑ *Alistipes* ↑ *Clostridiales* ↑ *Christensenellaceae* ↑ *Ruminococcaceae* ↓ *Faecalibacterium*, ↓ *Agathobacter*, ↓ *Bacteroides*, ↓ *Blautia*, ↓ *Lachnospira*.	↑ beta diversity ↔ alpha diversity	↓ butyrate ↓ acetate	N/A	Post-weight-restoration patients: ↔ gut microbiome composition ↔ beta diversity ↔ SCFA levels	Alpha diversity was not associated with BMI and EDE-Q score changes.
Schulz et al. 2021 [38]	L	19 AN (BMI 15.76 ± 2.03 kg/m^2^) 20 HC (BMI 20.31 ± 2.35 kg/m^2^) N/A PSY	16S rRNA	↑ *Anaerostipes* ↓ *Romboutsia*	↔ alpha diversity	N/A	AN patients at admission had a BDI 2 mean score of 22.68, compared to at discharge mean of 17.0, compared to the HCs’ mean of 5.65.	Post-weight-restoration patients (compared to HCs): ↑ Firmicutes ↑ Lachnospiraceae ↑ *Fusicatenibacter* ↓ *Romboutsia*	A higher abundance of unclassified Lachnospiraceae in AN patients at admission is associated with a shorter duration of treatment.
Yuan et al. 2022 [39]	CS	30 AN (BMI 14.92 ± 2.54 kg/m^2^) 30 HC (BMI 20.89 ± 2.14 kg/m^2^) N/A PSY	16S rRNA	↑ Lachnospiraceae ↑ *Eubacterium hallii* ↓ Ruminococcaceae ↓ *Faecalibacterium* ↓ *Subdoligranulum*	↑ beta diversity ↔ alpha diversity	N/A	AN patients had an HAMD mean score of 5, compared to the HCs’ mean of 2.	N/A	Negative correlation between BMI and Bacteroidota, *Bacteroides*, and Proteobacteria. Positive correlation between BMI and *Subdoligranulum*, Firmicutes.

**Table 3:** Significant changes in the relative abundance of gut microbiota and bacterial metabolites in AN patients compared to HCs within each of the included studies (n = 14). Symbols represent increased (↑), decreased (↓), or unchanged (↔).

	Armougom	Borgo	Fan	Fouladi	Hanachi	Kleiman	Mack	Million	Monteleone	Morita	Morkl	Prochazkova	Schulz	Yuan
**Phylum**														
Bacterioides	↔		↓				↓		↑					
Firmicutes	↔	↓					↑		↓					
Actinobacteria			↓				↑		↑					
Verrucomicrobia							↑							
Proteobacteria		↑												
**Family**														
Coriobacteriaceae						↑			↓		↑			
Ruminococcaceae		↓	↑											↓
Enterobacteriaceae		↑												
Peptostreptococcaceae														
Oxalobacteraceae									↓					
**Genus**														
*Ruminococcus*		↓			↑							↑		
*Roseburia*		↓	↓		↓		↓							
*Clostridium*		↓	↑			↓	↑			↓		↑		
*Bacterioides fragilis*										↓				
*Streptococcus*										↓				
*Lactobacillus*	↔		↑					↓		↓				
*Eubacterium*					↓									↑
*Anaerostipes*						↓							↑	
*Turicibacter*					↑									
*Anaerotruncus*					↑									
*Salmonella*					↑									
*Klebsiella*					↑									
*Methanobrevibacter*	↑	↑					↑	↑						
*Gemmiger*														
*Bifidobacterium*				↓										
*Faecalibacterium*				↓		↓						↓		↓
*Christensenella*			↑									↑		
*Escherichia*								↑						
*Weissella*									↑					
*Coprococcus*									↑					
*Parabacterioides*									↓					
*Alistipes*												↑		
*Agathobacter*												↓		
*Lachnospira*												↓		↑
*Romboutsia*													↓	
**Bacterial metabolites**														
Acetate										↓		↓		
Butyrate		↓					↓					↓		
Propionate		↓								↓				
Iso-Valerate		↔					↑							
Iso-butyrate		↔					↑							

**Table 4:** Summary of findings in AN patients with psychiatric comorbidities: significant changes in the gut microbiome (n = 4).

References	Significant Changes in Gut Microbiome Composition among AN Patients with Psychiatric Comorbidities (Depression or Anxiety)
Borgo	Negative correlation between BDI depression score and *Clostridium* spp. Negative correlation between faecal butyrate concentration and depression and anxiety scores.
Kleiman	Greater levels of depression were negatively associated with alpha diversity.
Monteleone	Positive correlation with BSI scores: *Coprococcus*, *Clostridium IV*, *Roseburia*, *Termsporobacter*.
Morkl	Negative correlation between alpha diversity and levels of depression.

## Data Availability

Not applicable.
